# Music@Home–Retrospective: A new measure to retrospectively assess childhood home musical environments

**DOI:** 10.3758/s13428-024-02469-2

**Published:** 2024-08-05

**Authors:** Nicholas Kathios, Kelsie L. Lopez, Laurel Joy Gabard-Durnam, Psyche Loui

**Affiliations:** 1 Department of Psychology, College of Science, Northeastern University, Boston, MA USA; 2 Department of Music, College of Arts, Media, and Design, Northeastern University, Boston, MA USA

**Keywords:** Early life experiences, Childhood, Music, Retrospective reporting, Scale validation

## Abstract

Early home musical environments can significantly impact sensory, cognitive, and socioemotional development. While longitudinal studies may be resource-intensive, retrospective reports are a relatively quick and inexpensive way to examine associations between early home musical environments and adult outcomes. We present the Music@Home–Retrospective scale, derived partly from the Music@Home–Preschool scale (Politimou et al., 2018), to retrospectively assess the childhood home musical environment. In two studies (total *n* = 578), we conducted an exploratory factor analysis (Study 1) and confirmatory factor analysis (Study 2) on items, including many adapted from the Music@Home–Preschool scale. This revealed a 20-item solution with five subscales. Items retained for three subscales (Caregiver Beliefs, Caregiver Initiation of Singing, Child Engagement with Music) load identically to three in the Music@Home-–Preschool Scale. We also identified two additional dimensions of the childhood home musical environment. The Attitude Toward Childhood Home Musical Environment subscale captures participants’ current adult attitudes toward their childhood home musical environment, and the Social Listening Contexts subscale indexes the degree to which participants listened to music at home with others (i.e., friends, siblings, and caregivers). Music@Home–Retrospective scores were related to adult self-reports of musicality, performance on a melodic perception task, and self-reports of well-being, demonstrating utility in measuring the early home music environment as captured through this scale. The Music@Home–Retrospective scale is freely available to enable future investigations exploring how the early home musical environment relates to adult cognition, affect, and behavior.

## Introduction

Developmental plasticity allows for environmental experiences to shape brain and behavior (McLaughlin & Gabard-Durnam, 2022; Werker & Hensch, 2015). In particular, heightened plasticity during early development facilitates lasting effects of experiences from this time period throughout the lifespan (Gabard-Durnam & McLaughlin, 2020; Keuroghlian & Knudsen, 2007; Reh et al., 2020). As an example in the domain of speech and language, classic work has shown that children’s heightened sensitivity to sounds in their environment allows them to develop lifelong expertise for the phonology of their native language(s) (Kuhl et al., 2006; Ortiz-Mantilla et al., 2016; Werker & Tees, 2002). Another key element of the early auditory home environment comes from exposure to music. Survey, interview, and in-home recording studies have shown that children routinely engage in informal musical activities (such as spontaneous singing, being sung to, listening to music, or playing with musical toys) starting in infancy and extending throughout childhood (Custodero & Johnson-Green, 2003; Fancourt & Perkins, 2018a; Ilari, 2005; Mendoza & Fausey, 2021; Yan et al., 2021; Young, 2008). Given the prevalence of music in the home environment during a time of heightened brain plasticity, many have asked whether and how such music exposure might have long-term impacts beyond childhood.

Research investigating the associations between formal musical training during childhood (i.e., instrumental instruction) and behavior over development and into adulthood suggests that such associations may also exist for early, informal musical engagement in the home environment. Although there is debate about whether and how musical training transfers to skills in other domains (see Sala & Gobet, 2020 and Bigand & Tillman, 2022), converging evidence from several longitudinal studies, such as the LongGold project (https://longgold.org/; Müllensiefen et al., 2022; Müllensiefen et al., 2015), suggests that musical training may benefit cognitive abilities throughout middle childhood and into adolescence (for a meta-analysis, see Román-Caballero et al., 2022). Specifically, musical training during this window leads to improvements in inhibitory control (Hennessy et al., 2019) and working memory (Lippolis et al., 2022), as well as greater activation of brain areas supporting inhibition (Sachs et al., 2017) and brain structure volumes of auditory and motor areas (Hyde et al., 2009). Moreover, several studies have shown that early musical training is associated with improved adult working memory (Tierney et al, 2015; Bailey and Penhune, 2010) and white matter volume in adulthood (Steele et al., 2013, Bailey et al., 2014). Together, these findings suggest that associations between formal musical training and behavior may extend to musical behaviors in children’s home environments. Indeed, studies have shown the immediate importance of musical engagement in the home in developmental samples, finding positive impacts on language (Papadimitriou et al., 2021) and auditory perception (for a review, see Putkinen et al., 2013). How these impacts of the informal music environment in the home persist into adulthood, however, remains to be tested.

The childhood home musical environment may also have impacts beyond perceptual and cognitive abilities on socioemotional functioning. Specifically, studies with children have identified positive associations between shared musical engagement, such as joint music-making, and prosocial behavior toward other children (Kirschner & Tomasello, 2010; Schellenberg et al., 2015). Investigations of shared musical behavior in the home musical environment between siblings or a child and their caregiver have shown similar positive relationships with prosocial behavior (Cirelli et al., 2020a, 2020b; Williams et al., 2015) and child–caregiver connectedness (Steinberg et al., 2021; Persico et al., 2017). Importantly, music also plays an important affective role in childhood, with studies showing that children’s engagement with music is associated with better emotional and self-regulation (Williams et al., 2015; Winsler et al., 2011). Together, these studies suggest the importance of measuring social musical behavior in the home environment and point to the potential relevance of such behavior in adult socioemotional outcomes.

Importantly, few measures of the home musical environment currently exist (e.g., Ruth & Müllensiefen, 2021), and most are caregiver reports intended for infants, preschoolers, or children (Brand, 1985; Politimou et al., 2018; Zdzinski, 2013). Within the extant scales, both the Home Musical Environment Scale (HOMES; Brand, 1985) and Parental Involvement–Home Environment in Music scale (PI-HEM; Zdzinski, 2013) include reporter attitudes as a dimension of the home musical environment, emphasizing the importance of measuring attitudes toward musical environments in such scales. However, existing questionnaires present challenges in linking the childhood home musical environment to longer-term outcomes including adult behavior, because such associations could only be detected through a longitudinal study following a cohort from childhood to adulthood, or by administering the report to the childhood caregivers of adult participants. As both of these options are relatively impractical due to their resource-intensiveness, the ability to link the childhood home music environment to adult behavior hinges on the development of a retrospective self-report measure of the childhood home musical environment. Further, self-report measures are a relatively quick and inexpensive way to collect large amounts of data and generate hypotheses for future prospective studies (Hess, 2004).

### The present study

To address this important gap in measurement, here we present the Music@Home–Retrospective scale, an instrument aimed at capturing adult self-reports of their home musical environment during middle childhood. This scale captures the same dimensions of the home musical environment as the Music@Home–Preschool scale (Parental Beliefs, Child Engagement with Music, Parent Initiation of Musical Behavior, and Breadth of Musical Exposure). We also developed items to index individuals’ engagement in social music behavior given the empirical work showing relationships between shared musical engagement in the home and socioemotional profiles (Williams et al., 2015; Steinberg et al., 2021; Persico et al., 2017). Additional items were created to measure attitudes toward their home musical environment, consistent with existing scales for self-report during childhood (Brand, 1985; Zdzinski, 2013). We validate the Music@Home–Retrospective scale psychometrically and demonstrate its utility by linking scores to tests of adult music perception and self-reports of current (adult) music usage and mental health. We hope that the Music@Home–Retrospective scale offers aa effective, freely available tool for probing long-term associations between childhood music engagement and brain and cognitive health as well as social and emotional well-being.

We focus this questionnaire on measuring the home musical environment during middle childhood (6–12 years; Mah & Ford-Jones, 2012). Although the original Music@Home questionnaires (Infant and Preschool versions) index the home musical environment between the ages of 3 months and 5.5 years, we ask participants to report on their experiences in middle childhood. We include these ages because adults typically cannot recall autobiographical memories from the first few years of life due to infantile amnesia (Nelson & Fivush, 2004; Peterson et al., 2011; Rubin, 2000), and 12 years old is typically considered the end of middle childhood (Bertram & Pascal, 2002; Le Métais, 2003, Leseman et al., 2017; Mah & Ford-Jones, 2012).

## Study 1: Exploratory factor analysis

### Methods

#### Developing items for the Music@Home–Retrospective Scale

The goal of Study 1 was to identify a factor structure for items produced for the Music@Home–Retrospective scale with an exploratory factor analysis (EFA). A total of 31 items were tested for this EFA. Seventeen of these items were adapted from the Music@Home–Preschool (intended for children aged 2 to 5.5 years) scale to measure retrospective reports of the home musical environment in childhood (6 to 12 years). The Music@Home–Preschool scale consists of a total of 17 items; however, one item (“My child was deliberately sung to/exposed to music whilst in the womb”) was excluded from the Music@Home–Retrospective scale as it did not pertain to childhood experiences. Another item (“I do not feel comfortable singing to my child in public or when others are around”) that was excluded from the original scale following poor psychometric performance (see Politimou et al. 2018) was reintroduced in modified form into the Music@Home–Retrospective to index exposure to caregiver singing (bringing the total number of items included from the Music@Home–Preschool scale back to 17 items). The original items were edited to read from the point of view of the participant, rather than from the point of view of the caregiver and were now presented in the past tense (apart from questions prompting current attitudes toward childhood musical experiences).

An additional 14 items were included to capture shared musical engagement in the home and attitudes toward their home musical environment (see Appendix 1 for the list of all items originally administered to the EFA sample). Items capturing attitude focused on attitudes toward the childhood home musical environment as both adolescents (retrospective attitude, i.e., adults reporting their attitude during adolescence toward the childhood home musical environment) and adults (current attitude, i.e., adults reporting their current attitudes toward the childhood home musical environment) to capture attitudes following the middle childhood time frame. The additional items added to the Music@Home–Retrospective scale were pulled from an unvalidated questionnaire used in a previous music study with adult retrospective reports of childhood musical experiences (Gabard-Durnam et al., 2018).

With these 31 initial items, the questionnaire was divided into three sections, which reflected the goals of indexing (1) the childhood home musical environment, (2) retrospective adolescent attitude toward the childhood home musical environment, and (3) current (adult) attitudes toward the childhood home musical environment. The first section prompted experiences of music in the household as a child (ages 6–12 years), with 17 items from the Music@Home–Preschool and three additional items. In this initial section, we aimed to replicate four factors from the Music@Home–Preschool scale: Parental Beliefs, Child Engagement with Music, Parent Initiation of Musical Behavior, and Breadth of Musical Exposure. The second section included three items to measure attitudes toward childhood music in the household as an adolescent (ages 13–18). The third section introduced eight items assessing current attitudes toward music in the childhood household. For consistency with Politimou et al. (2018), a seven-point response scale was used for the Music@Home–Retrospective scale, ranging from “completely disagree” (1) to “completely agree” (7).

#### Participants

A total of 299 English-speaking adult (18–66 years old) participants from the United States completed Study 1. Nine participants were removed prior to data analysis because of missing data, specifically in response to the initial 34 Music@Home–Retrospective items (see Table 1 for demographic information of the 290 participants included in our Study 1 analyses). Participants were recruited from a sample who participated in a previous online study run by members of our lab (Kathios et al., 2024) through the online software Prolific. Participants were recontacted in order to use a series of questionnaires that were previously collected in this sample. All participants from that sample were recontacted with the opportunity to participate in the current online study.

**Table 1:** Study 1 sample demographics

Demographic	Study 1 sample (*n* = 290)
Age	
Mean (SD)	35.92 years (11.74)
Range	18–66 years
Sex	
Male	126 (43.44%)
Female	163 (56.21%)
No response	1 (0.34%)
Gender	
Man	126 (43.44%)
Woman	159 (54.83%)
Non-binary/Other	4 (1.38%)
Choose not to disclose	1 (0.34%)
Race/Ethnicity	
White	194 (66.9%)
Black or African American	25 (8.62%)
Native American	1 (0.34%)
Asian	28 (9.66%)
Native Hawaiian or Pacific Islander	0 (0%)
Hispanic or Latino	15 (5.17%)

Demographic information for the Study 1 sample

#### Materials and procedure

Participants completed the 34-item Music@Home–Retrospective scale as part of a larger questionnaire battery in Study 1. Participants completed all questionnaires on the Qualtrics online survey tool, which took 45 minutes on average.

#### Analysis plan

Exploratory factor analysis (EFA) on the Music@Home–Retrospective scale was performed using the psych::fa function within the *psych 2.2.9* package (Revelle, 2024) in R version 4.2.1 (2022-06-23). The goals of the EFA were to (1) identify the existence of a general factor, (2) identify the factor structure, and (3) reduce items to include only those most relevant for retrospectively assessing the childhood home musical environment.

We followed similar statistical procedures carried out by Politimou et al. (2018) to perform the exploratory factor analysis. We first tested a bifactor model to indicate the presence of a general factor using the McDonald coefficient omega test (McDonald, 1999; threshold for general factor: ωh > 0.6). In bifactor models, items uniquely contribute to both the general score (accounting for all items in the scale) and to a factor. We chose to test a bifactor model over a higher-order model for similar reasons as Politimou et al. (2018): unlike higher-order models, bifactor models allow for the existence of factors independent of the general factor through assessment of items’ direct associations with the general factor. This best enables an examination of the relationships with specific factors of our scale, which is a goal of the Music@Home–Retrospective (as it is with the Music@Home questionnaire). Because the McDonald coefficient omega test requires a factor solution, we initially tested the fit of a six-factor solution based on six dimensions (four dimensions from the original Music@Home–Preschool scale, with the addition of current and adolescent attitudes). To determine an initial factor solution on which to conduct item reduction, we employed maximum likelihood estimation factor extraction. We evaluated extracted factors using parallel analysis, Kaiser’s criterion, inspection of the scree plot, and Velicer’s minimum average partial (MAP) criterion. To reduce items, we used the Schmid–Lieman factor analysis solution with maximum likelihood estimation and oblique rotation, as determined by Politimou et al. (2018) for use on this scale. We then removed individual items based on the following criteria: not loading (<0.2) onto the general factor, high uniqueness value (>0.7), two factor loadings > 0.2 for a single question, or not loading (<0.2) onto any factor. While there is not an agreed-upon procedure for item removal criteria across the scale validation literature, we chose for our criteria to mirror the cutoffs used in Politimou et al. (2018) in order to be consistent and facilitate comparison between the two questionnaires. This process was completed iteratively, starting from the determination of the factor solution according to the specified factor extraction techniques above. When there were discrepancies between the number of factors identified by these techniques, we chose the model with the lowest root mean square error of approximation (RMSEA) on which to perform item reduction (Hu & Bentler, 1999, suggest a threshold “cutoff value close to .06” as indicating “a relatively good fit between the hypothesized model and the observed data”). During each iteration, items were removed, and a new number of factors was determined using the factor extraction methods outlined above. This process was repeated until no items violated this removal criteria. An additional McDonald coefficient omega test was performed following item reduction to indicate the presence of a general score for the final factor structure.

### Results

#### Exploratory factor testing

A preliminary exploratory factor analysis on six factors using Kaiser’s criterion (Dinno, 2009), visual inspection of the screeplot (Cattell, 1966), and Velicer’s minimum average partial (MAP; Velicer, 1976) criterion all converged on a six-factor solution (RMSEA for this model was .061). We then used this factor solution to test for the presence of a general factor using a McDonald coefficient omega test, which suggested the presence of a general factor and provided evidence that this model is bifactor (ω_h_ = 0.69).

To account for this general factor, we completed our factor extraction and item reduction procedures on the residual matrix of a factor analysis with only one factor. Parallel analysis on the residual matrix indicated four factors, while Velicer’s MAP indicated five factors. However, visual inspection of the scree plot using Kaiser’s cutoff determined seven factors as the best fit, which yielded the lowest RMSEA value (0.055). As a result, we continued item reduction on this seven-factor structure solution. Following this initial item removal step, factor extraction methods were run again to determine a new number of factors and repeated until all items that violated the predetermined criteria were removed and a final factor structure was revealed.

**Fig. 1:**
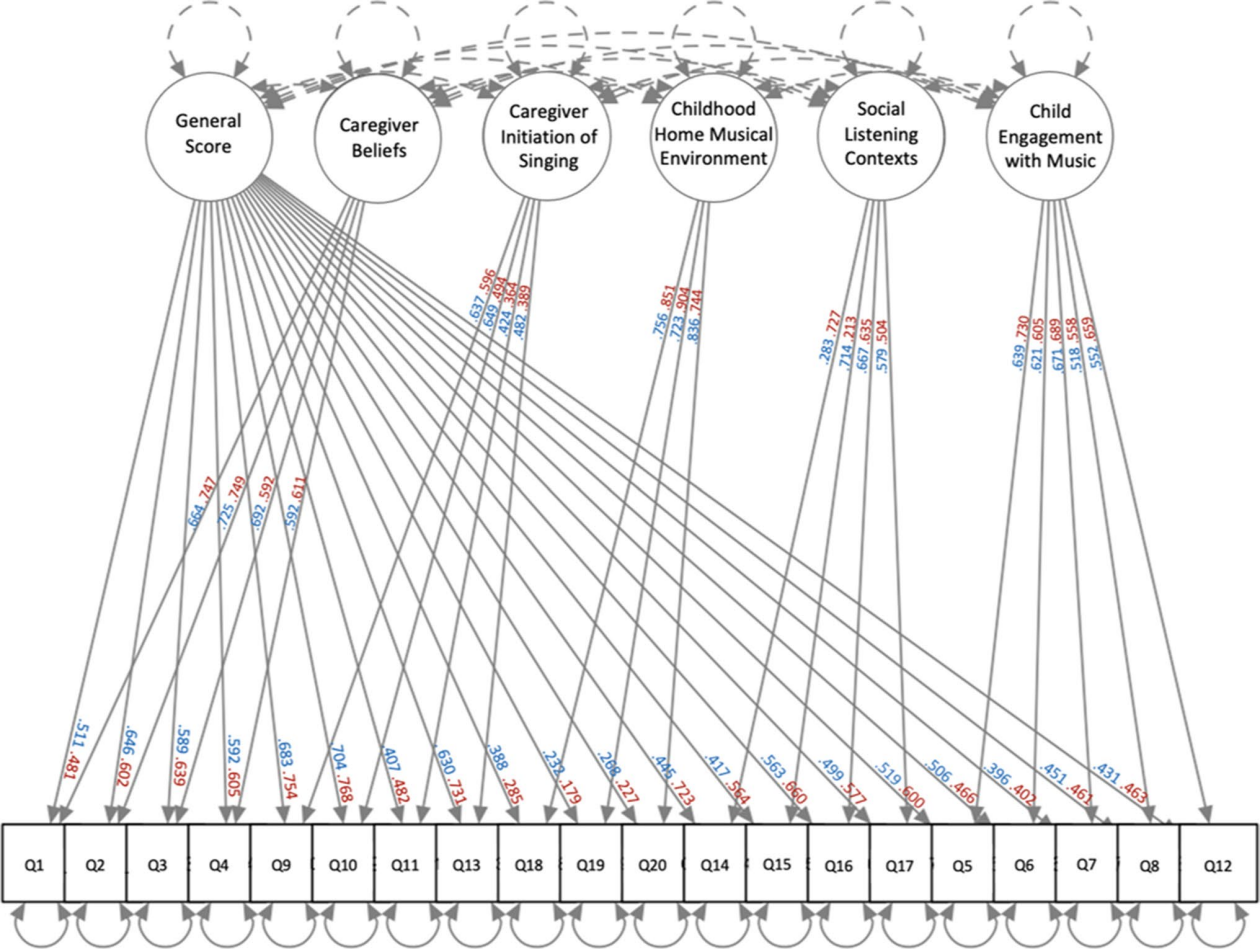
Factor analysis of Music@Home–Retrospective scale. Straight lines indicate item loadings onto both the general score and their respective factor. Dotted lines indicate restrictions applied to the confirmatory factor analysis model: residual variances (shown with curved lines) of factors were set to 1, and factors were set to be uncorrelated (shown with lines in between factors; Chen et al., 2006). Blue loadings correspond to Study 1 (EFA) whereas red loadings correspond to Study 2 (CFA). Refer to Appendix 2 for the items on the Music@Home–Retrospective scale along with scoring instructions

The EFA with the iterative item reduction procedure revealed a final factor structure with five factors in addition to a general factor, with 20 items out of the initial 31 retained (see Fig. 1 for EFA factor loadings). Three iterations of the item reduction procedure were run before reaching a final solution with five factors, with items removed based on the predetermined criteria: three items for not loading onto the general score, seven items for two factor loadings > 0.2 for a single question, and one item for both having two factor loadings > 0.2 and having a high uniqueness value greater than 0.7. A McDonald coefficient omega test run on the final factor structure again indicated the presence of a general factor and a bifactor model (ωh = 0.62). This general factor provides a composite score that measures the role of music in the childhood home environment.

Our next step was identifying names for the five identified factors. Three of our factors contained items that loaded identically in our adult sample as they did for the original Music@Home–Preschool scale (Parental Beliefs, Parent Initiation of Musical Behavior, and Child Engagement with Music). For this reason, we named these factors according to their complement on the Music@Home–Preschool scale: Caregiver Beliefs, Caregiver Initiation of Singing, and Child Engagement with Music, respectively. It is important to note that, although all items from the Music@Home–Preschool Scale Parent Initiation of Musical Behavior were included in our testing, only items referring to caregiver singing were maintained across our item reduction procedures. For this reason, although the items retained for the Music@Home–Retrospective loaded identically to those from the Music@Home–Preschool Parent Initiation of Musical Behavior factor, we opted to label this factor Caregiver Initiation of Singing. The two factors which contained items not originating from the Music@Home–Preschool measured adult attitudes toward individuals’ home musical environment (labeled the Attitude Toward Childhood Home Musical Environment factor) and the extent to which individuals listened to music with others at home as children (labeled the Social Listening Contexts factor). No items probing retrospective attitudes toward childhood music during adolescence survived item reduction.

### Discussion

The primary goal of Study 1 was to determine the underlying factor structure of the 34 items produced to retrospectively measure the childhood home musical environment. Exploratory factor analyses and subsequent item removal on a sample of 290 adult participants revealed a five-factor solution with 20 items retained. The five identified factors retrospectively measured participants’ caregivers’ beliefs regarding music (Caregiver Beliefs), the degree to which participants were sung to (Caregiver Initiation of Singing), how often they made music at home (Child Engagement with Music), how often they listened to music with others (Social Listening Contexts), and their current attitudes toward their childhood home musical environment (Attitude Toward Childhood Home Musical Environment). Finally, McDonald coefficient tests both before and following item removal indicated that this factor solution is bifactor with a general construct that measures participants’ overall recollection of their childhood home musical environment. Building on these results, our next goals in Study 2 were to (1) confirm this five-factor bifactor solution in an independent sample with confirmatory factor analysis, (2) establish the test–retest reliability of the Music@Home–Retrospective scale, and (3) demonstrate the scale’s utility by testing associations across measures of adult musicality and socioemotional well-being.

## Study 2: Confirmatory factor analysis

### Methods

#### Participants

A total of 288 English-speaking adult (18–55 years old) participants from the United States completed Study 2 (see Table 2 for demographic information). Participants were recruited from a sample who participated in a previous online study through Prolific (separate from the one from which we drew participants for the EFA; Kathios et al., 2023). Forty-six participants were enrolled in both Study 1 and Study 2. They were removed from CFA analyses but included in analyses measuring the test–retest reliability of the Music@Home–Retrospective scale over 4 months. All 288 participants were included in analyses evaluating associations of the Music@Home–Retrospective scale to current adult behavior and mental health.

**Table 2:** Study 2 sample demographics

Demographic	Entire study 2 sample (*n* = 288)	Test–retest sample (*n* = 46)
Age		
Mean (*SD*)	34 (9.11)	35 (8.59)
Range	18–55 years	22–54 years
Sex		
Male	145 (50.35%)	21 (45.65%)
Female	141 (48.96%)	24 (52.17%)
No response	2 (0.69%)	1 (2.17%)
Gender		
Man	146 (50.69%)	21 (45.65%)
Woman	136 (47.22%)	23 (50%)
Non-binary/Other	4 (1.39%)	1 (2.17%)
Choose not to disclose	2 (0.69%)	1 (2.17%)
Race/Ethnicity		
White	196 (68.06%)	30 (65.22%)
Black or African American	27 (9.38)%	4 (8.7%)
Native American	0 (0%)	0 (0%)
Asian	22 (7.64%)	4 (8.7%)
Native Hawaiian or Pacific Islander	0 (0%)	0 (0%)
Hispanic or Latino	16 (5.56%)	3 (6.52%)
Other/Mixed Race/Ethnicity	25 (8.68%)	4 (8.7%)
No response	2 (0.69%)	1 (2.17%)

Demographic information for the entire Study 2 sample, as well as only those included in the test–retest analysis

#### Materials

In addition to the Music@Home–Retrospective scale, participants completed a battery of other relevant questionnaires to explore associations between the proposed scale and relevant existing scales. Specifically, we included the Social Reward Questionnaire (SRQ; Foulkes et al., 2014) to examine associations between the Music@Home–Retrospective scale and non-musical social reward in adulthood. Participants completed the sociability and prosocial interactions subscales of the adult SRQ, consisting of eight items out of the original 23 items in the scale. To examine associations between the Music@Home–Retrospective scale and music perception abilities, participants also completed the Musical Ear Test (MET; Wallentin et al., 2010) for rhythmic and melodic music perception. For the MET, participants are asked to indicate whether two short musical excerpts are the same or different. They are assessed on excerpts that test both melodic discrimination and rhythmic discrimination, split into two blocks. Within each block, “same” and “different” trials each occur 50% of the time and are randomized throughout. In addition, participants completed the 34-item Absorption in Music Scale (AIMS; Sandstrom & Russo, 2013) to measure the extent to which they are drawn into the music while engaging in music listening. Finally, to relate the childhood home musical environment to current adult music use and mental health, we measured adult music usage using the healthy and unhealthy subscales of the Healthy-Unhealthy Music Scale (HUMS; Saarikallio et al., 2015; validated in adolescents but still commonly used in adult samples), trait anxiety using the State-Trait Anxiety Inventory (STAI; Spielberger et al., 1970), and resiliency using the Connor-–Davidson Resilience Scale (CD-RISC-10; Campbell-Sills & Stein, 2007). The HUMS is a self-report measure of 13 items which asks participants to rate how much they engage in typically healthy (e.g., “Music helps me to relax”) and unhealthy (e.g., “Music gives me an excuse not to face up to the real world”) uses of music, resulting in separate measures of the degree of healthy and unhealthy music usage. The STAI is a 40-item measure of state and trait anxiety, but participants only completed the 20-item trait anxiety subscale. Finally, the CD-RISC-10 is a 10-item self-report measure of resilience, or adaptability and flexibility in stressful contexts.

Because participants were recruited from a sample who participated in a previous online study in our lab, we drew from the following previously collected survey scores to further establish associations with the Music@Home–Retrospective scale. The Goldsmiths Musical Sophistication Index (Gold-MSI; Müllensiefen et al., 2014) was included as a measure of musical training and sophistication, broken down into four scores: Active Engagement, Perceptual Abilities, Musical Training, Singing Abilities, Emotion, and Musical Sophistication. Additionally, we used scores from the 20-item Barcelona Music Reward Questionnaire (BMRQ; Mas-Herrero et al., 2013), a measure of individual differences in music-reward sensitivity (on four dimensions: Mood Regulation, Emotion Evocation, Social Reward, Music Seeking, and Sensorimotor). To include the Absorption subscale from the extended BMRQ (eBMRQ; Cardona et al., 2022) in this sample (and calculate overall eBMRQ scores), the four items that comprise the Absorption subscale were pulled from the Absorption in Music scale. Participants completed the experiment on the Qualtrics online survey tool, which took 55 minutes on average.

#### Procedure

Participants were initially directed to the online behavioral experiment software Gorilla to complete the MET (Correia et al., 2022) and then sent to the Qualtrics online survey tool to complete the psychometric survey battery.

#### Analysis plan

Confirmatory factor analysis (CFA) on the Music@Home–Retrospective scale was performed using the lavaan::cfa function within the lavaan 0.6.15 package (Rosseel, 2012) in R version 4.2.1 (2022-06-23). The confirmatory fit of the model to the data was tested using the statistical tests that were employed previously in Politimou et al. (2018): RMSEA, Bentler’s comparative fit index (CFI), standardized root mean square residual (SRMR), and Tucker–Lewis Index (TLI). Model fit was evaluated based on stringent thresholds from Hu and Bentler (1999), with “a cutoff value close to 0.06 for RMSEA”, “close to 0.95 for CFI,” “close to 0.08 for SRMR,” and “close to 0.95 for TLI” , which demonstrate “a relatively good fit between the hypothesized model and the observed data.” In addition, the general score and five factors were set to be uncorrelated, while residual variances of factors were indicated as 1 (as done in Politimou et al., 2018, and determined as best for bifactor models by Chen et al., 2006). Finally, remaining items in the CFA were expected to meet a 0.2 loading threshold for their respective factors and the general factor (based on the threshold in Politimou et al. (2018).

A subsample of 46 participants in Study 2 had also completed the Music@Home–Retrospective scale in Study 1. These participants were used to assess test–retest reliability (about 4 months passed between Study 1 and Study 2) of the Music@Home–Retrospective scores using intraclass correlations (ICCs) generated from the psych::ICC function within the psych 2.2.9 package in R. ICC thresholds were determined based on criteria set forth by Cicchetti (1994) for test–retest reliability of psychometric questionnaires: fair, .40 ≤ values ≤ .59; good, .60 ≤ values ≤ .74; and excellent, values ≥ .75.

Finally, an additional goal of Study 2 was to evaluate associations of the childhood home music environment assessed in the Music@Home–Retrospective scale with other relevant measures of adult behavior and mental health using the MET, Gold-MSI, eBMRQ, SRQ, HUMS, STAI, and CD-RISC. This approach was in lieu of construct validity analyses because there currently are no validated retrospective scales that can be used to directly assess the validity of the proposed scale. Bivariate correlations for associations between scales were assessed using the stats::cor.test function within the stats 4.2.1 package in R, while linear regressions were run using the stats::lm function. All questionnaires were standardized in linear regression models for comparability across measures. For these analyses, all 288 participants were included.

### Results

#### Confirmatory factor testing

The CFA confirmed the bifactor model with five factors in addition to the general factor, as determined by the EFA in Study 1. The RMSEA, Bentler’s comparative fit index (CFI), standardized root mean square residual (SRMR), and Tucker–Lewis Index (TLI) statistical tests revealed a relative good fit of the model to the data based on Hu and Bentler (1999) thresholds (RMSEA: 0.064; CFI: 0.960; SRMR: 0.063; TLI: 0.950; also consistent with less stringent thresholds; Browne & Cudeck, 1993). All items met the 0.2 loading threshold for their respective factors and the general factor (see Fig. 1 for CFA loadings), with the exception of item 19 (“I have negative memories of the music I heard in the home as a child.”), which had a loading of 0.179 onto the general score. However, we did not remove this question due to a strong loading (0.904) onto its respective factor.

Additionally, no Music@Home–Retrospective subscale scores were significantly correlated with age at retrospective reporting, indicating that there was no biased recollection of the home environment as a function of age within the sample (all *p* > .05, *n = *287; one participant was missing data for age).

#### Test–retest reliability

To assess test–retest reliability for the Music@Home–Retrospective scale, we calculated intraclass correlations (ICCs) with the 46 participants who filled out the scale in Studies 1 and 2 (after about 4 months). Overall Music@Home–Retrospective Scores had excellent test–retest reliability (ICC = .84) based on thresholds put forth by Cicchetti (1994). The Caregiver Beliefs (ICC = .84), Caregiver Initiation of Singing (ICC = .84), and Social Listening Contexts (ICC = .85) factors also demonstrated excellent test–retest reliability, while Child Engagement with Music (ICC = .72) and Attitude Toward Childhood Music Environment (ICC = .64) demonstrated good test–retest reliability (see Table 3).

**Table 3:** Test–retest reliability

M@H-R subscales	Test–retest (ICC) over 4 months
Caregiver Beliefs	.84
Child Engagement with Music	.72
Caregiver Initiation of Singing	.84
Social Listening Contexts	.85
Attitude Toward Childhood Music Environment	.64
General	.84

Music@Home–Retrospective test–retest reliability measured via intra-class correlation (ICC) values between Study 1 and Study 2 (4-month delay) for 46 participants. The following thresholds were used: fair, .40 ≤ values ≤ .59; good, .60 ≤ values ≤ .74; and excellent, values ≥ .75 for test–retest reliability

#### Associations with adult behavior and mental health

A third goal of Study 2 was to evaluate associations of the Music@Home–Retrospective scale with other relevant measures in adulthood to demonstrate its utility. Specifically, we aimed to relate the childhood home musical environment to current musicality and socioemotional well-being.

**Fig. 2:**
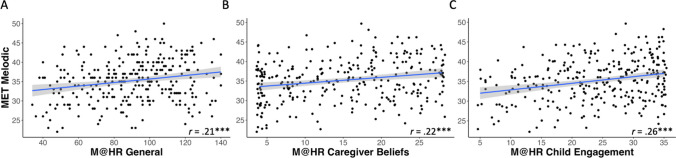
Scatterplots of bivariate correlations relating Musical Ear Test (MET) melodic score with Music@Home–Retrospective scale: **A** General score (*n* = 288); **B** Caregiver Beliefs (*n* = 288); **C** Child Engagement with Music (*n* = 288). Gray shading indicates the 95% confidence interval of the regression line. * *p* < .05, ** *p* < .01, *** *p* < .001

**Fig. 3:**
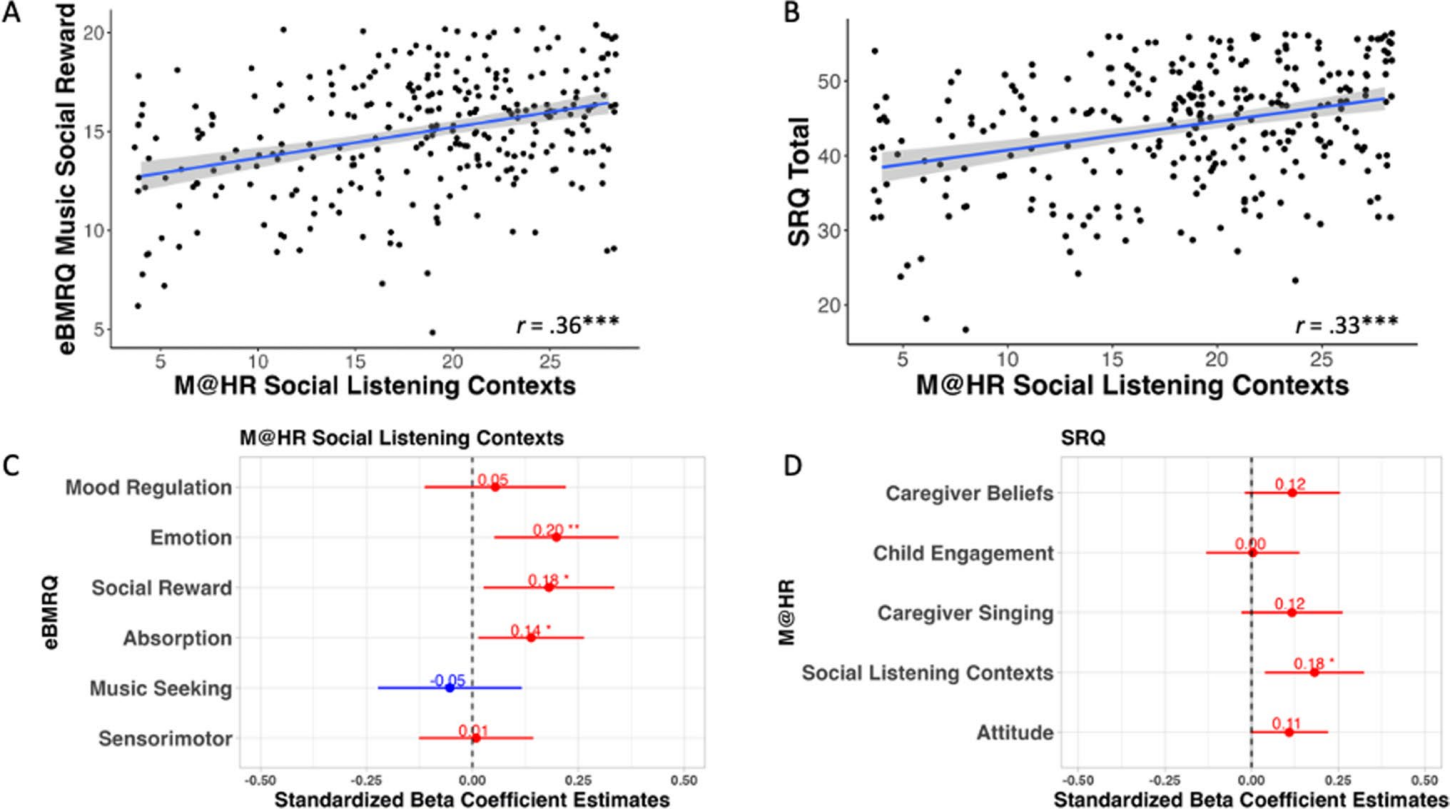
**A** Scatterplot of bivariate correlation relating Music@Home–Retrospective scale Social Listening Contexts and Extended Barcelona Music Reward Questionnaire (eBMRQ) Social Reward (*n* = 288). **B** Scatterplot of bivariate correlation relating Music@Home–Retrospective scale Social Listening Contexts and SRQ (*n* = 288). **C** Standardized beta coefficient estimates for eBMRQ subscales as predictors of Music@Home–Retrospective scale Social Listening Contexts. **D** Standardized beta coefficient estimates for Music@Home–Retrospective scale factors as predictors of SRQ scores. Gray shading indicates the 95% confidence interval of the regression line. * *p* < .05, ** *p* < .01, *** *p* < .001

First, associations between home musical environment and adult music perception abilities were tested by correlating Music@Home–Retrospective scale scores with MET task performance. The Music@Home–Retrospective general score (*r*(286) = .21, *p < *.001; Fig. 2A) and the Caregiver Beliefs (*r*(286) = .22, *p* < .001; Fig. 3B) and Child Engagement with Music (*r*(286) = .26, *p < *.001; Fig. 2C) subscale scores were all significantly positively correlated with MET melodic scores in adulthood. That is, the more that participants reported their caregivers believed music was important and the more they had engaged with music as children, the better their music perception abilities were as adults. This finding was specific to performance for melodic discrimination, as there were no associations between Music@Home–Retrospective subscales and MET rhythmic scores (all *p* > .05, *n* = 288).

Turning to self-reported measures of musical ability, training, and sophistication, all subscales of the Music@Home–Retrospective scale were significantly positively correlated with all scores on the Gold-MSI (all *p* < .01, *n = *288), with the exception of the Attitude Toward Childhood Music Environment subscale. This subscale was only positively correlated with the Active Engagement (*r*(286) = .14, *p = *.02), Perceptual Abilities (*r*(286) = .18, *p = *.002), and Emotion (*r*(286) = .17, *p = *.004) scores on the Gold-MSI (see Table 4).

**Table 4:** Gold-MSI score associations

	Gold-MSI scores					
M@H-R scores	Active Engagement	Perceptual Abilities	Musical Training	Singing Abilities	EmotionEvocation	Musical Sophistication
Caregiver Beliefs	.23***	.21***	.52***	.23***	.20***	.35***
Child Engagement with Music	.32***	.36***	.59***	.33***	.38***	.50***
Caregiver Initiation of Singing	.29***	.19**	.21***	.30***	.29***	.30***
Social Listening Contexts	.29***	.22***	.23***	.24***	.34***	.29***
Attitude Toward Childhood Music Environment	.14*	.18**	.08	.07	.17**	.10
General	.37***	.34***	.50***	.35***	.40***	.46***

Bivariate correlations relating Goldsmith Musical Sophistication Index (Gold-MSI) scores and Music@Home–Retrospective subscales in the Study 2 sample. * *p* < .05, ** *p* <.01, *** *p* <.001

Music@Home–Retrospective subscales were also compared to both musical and non-musical measures of reward in adulthood. With respect to musical reward in adulthood, significant positive associations were observed between the Music@Home–Retrospective General scale score and all subscales of the eBMRQ (all *p* < .001, *n* = 288). The Music@Home–Retrospective Attitude Toward Childhood Music Environment subscale was only positively correlated with the Mood Regulation (*r*(286) = .12, *p* = .04), Social Reward (*r*(286) = .18, *p* = .003), and Music Seeking (*r*(286) = .14, *p* = .02) eBMRQ subscales, while the Music@Home–Retrospective subscales Caregiver Beliefs, Child Engagement with Music, Caregiver Initiation of Singing, and Social Listening Contexts were each positively correlated with all subscales of the eBMRQ (all *p* < .05, *n* = 288; see Table 5). Linear regressions further probed the association between the Music@Home–Retrospective Social Listening Contexts subscale scores and adult musical social reward sensitivity as measured through the eBMRQ. This revealed that the Social Listening Contexts subscale scores were significantly associated with the eBMRQ Social Reward, Emotion Evocation, and Absorption subscales over and above other eBMRQ subscales (Social Reward: *B = *0.18, *t*(280) = 2.30, *p = *.02; Emotion: *B* = 0.20, *t*(280) = 2.67, *p* = .008; Absorption: *B = *0.14, *t*(280) = 2.19, *p = *.03; see Fig. 3C). That is, the more social listening reported from childhood, the more socially rewarding music was, the more emotion that music evoked, and the greater the tendency there was to absorb into music in adulthood. Moreover, with respect to non-musical social reward in adulthood, a second linear model revealed that the Social Contexts subscale score was also significantly positively associated with the Social Reward Questionnaire (SRQ) scores, over and above the other Music@Home–Retrospective subscales (*B* = 0.18, *t*(282) = 2.50,* p* = .01, see Fig. 3D and Table 6 for bivariate correlations between SRQ and Music@Home - Retrospective subscales). That is, the Music@Home–Retrospective Social Contexts subscale scores were especially closely associated with both musical and non-musical reward profiles in adulthood.

**Table 5:** eBMRQ subscale associations

	eBMRQ Subscales						
M@H-R subscales	Mood Regulation	Emotion	Social Reward	Absorption	Music Seeking	Sensorimotor	Total
Caregiver Beliefs	.21***	.18**	.29***	.19**	.23***	.12*	.27***
Child Engagement with Music	.30***	.34***	.38***	.21***	.26***	.29***	.38***
Caregiver Initiation of Singing	.30***	.27***	.33***	.22***	.25***	.27***	.36***
Social Listening Contexts	.29***	.37***	.36***	.30***	.26***	.26***	.40***
Attitude Toward Childhood Music Environment	.12*	.06	.18**	−.05	.14*	.06	.1
General	.35***	.36***	.44***	.27***	.33***	.29***	.44***

Bivariate correlations relating Extended Barcelona Music Reward Questionnaire (eBMRQ) subscales and Music@Home–Retrospective subscales in the Study 2 sample. * *p* < .05, ** *p* < .01, *** *p* < .001

**Table 6:** SRQ associations

M@H-R subscales	SRQ
Caregiver Beliefs	.26***
Child Engagement with Music	.20***
Caregiver Initiation of Singing	.31***
Social Listening Contexts	.33***
Attitude Toward Childhood Music Environment	.20***
General	.36***

Bivariate correlations relating the Social Reward Questionnaire (SRQ) and Music@Home–Retrospective subscales in the Study 2 sample. * *p* < .05, ** *p* < .01, *** *p* < .001

**Fig. 4:**
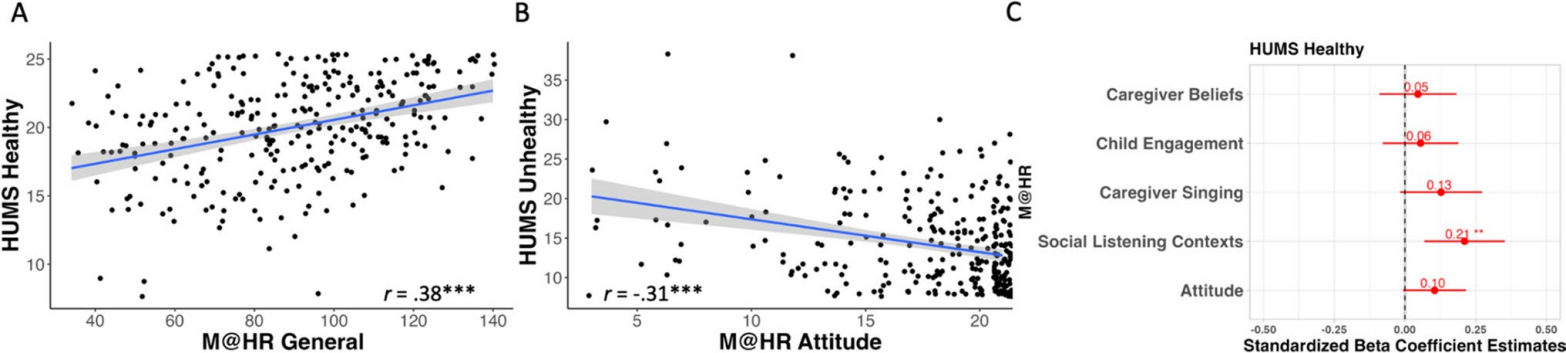
**A** Scatterplot of bivariate correlation relating Music@Home–Retrospective scale general score and Healthy-Unhealthy Music Scale (HUMS) Healthy subscale (*n* = 288). **B** Scatterplot of bivariate correlation relating Music@Home–Retrospective scale Attitude Toward Childhood Music Environment and Healthy-Unhealthy Music Scale (HUMS) Unhealthy subscale (*n* = 288). **C** Standardized beta coefficient estimates for Music@Home–Retrospective scale factors as predictors of HUMS Healthy subscale. Gray shading indicates the 95% confidence interval of the regression line. * *p* < .05, ** *p* < .01, *** *p* < .001

Next, Music@Home–Retrospective subscale scores and music usage profiles in adulthood were compared using the Healthy-Unhealthy Music Scale (HUMS). All subscales of the Music@Home–Retrospective scale were positively significantly correlated with healthy music usage scores on the HUMS (all *p* < .001, *n* = 288; see Fig. 4A). The Music@Home–Retrospective Attitude Toward Childhood Music Environment subscale was also negatively correlated with unhealthy music usage scores (e.g., a more positive attitude toward the childhood music environment was associated with less unhealthy music usage; *r*(286) = −.31, *p* < .001; see Fig. 4B). To differentiate which Music@Home–Retrospective subscales were most closely associated with healthy music usage over and above the other subscales, a linear regression was run with all subscales of the Music@Home–Retrospective scale included. This revealed that the Social Contexts subscale was a significant positive predictor of healthy music usage over and above the other subscale scores (*B* = 0.21, *t*(282) = 2.92, *p* = 0.003).

**Fig. 5:**
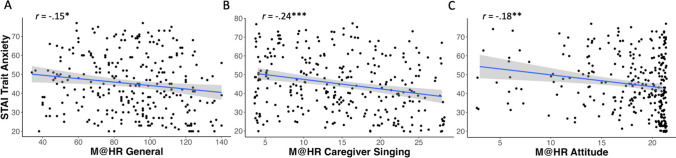
Scatterplots of State-Trait Anxiety Inventory (STAI) trait anxiety subscale bivariate correlations with Music@Home–Retrospective scale: **A** General score (*n* = 288); **B** Caregiver Initiation of Singing (*n* = 288); **C** Attitude Toward Childhood Music Environment (*n* = 288). Gray shading indicates the 95% confidence interval of the regression line. * *p* < .05, ** *p* < .01, *** *p* < .001

**Fig. 6:**
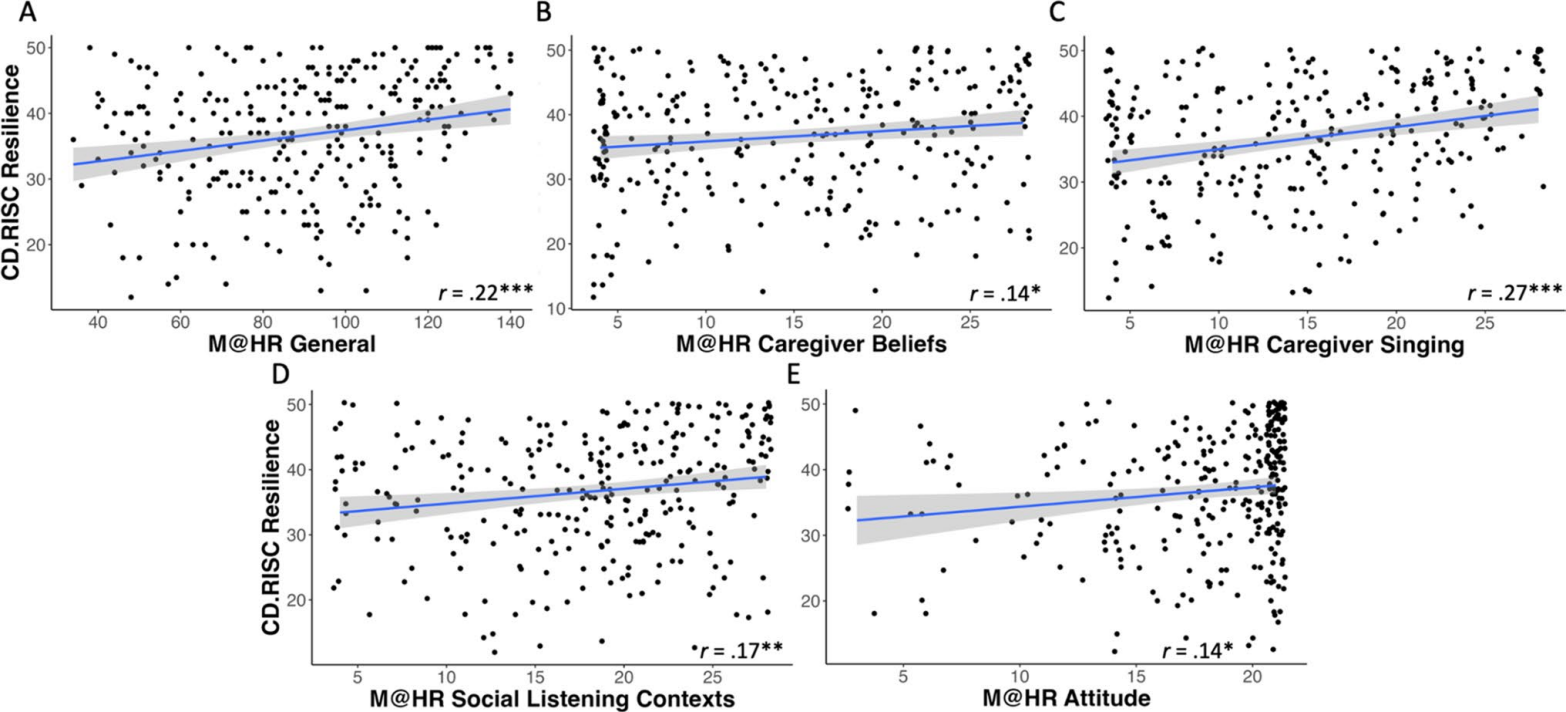
Scatterplots of Connor–Davidson Resilience Scale (CD-RISC) bivariate correlations with Music@Home–Retrospective scale: **A** General score (*n* = 288); **B** Caregiver Beliefs (*r*(286) = .14, *p* = .02); **C** Caregiver Initiation of Singing (*n* = 288); **D** Social Listening Contexts (*n* = 288); **E** Attitude Toward Childhood Music Environment (*n* = 288). Gray shading indicates the 95% confidence interval of the regression line. * *p* < .05, ** *p* < .01, *** *p* < .001

Finally, with respect to adult socioemotional well-being and mental health, the Music@Home–Retrospective scale scores were compared to adult trait anxiety and resilience measures. The Music@Home–Retrospective general score was significantly negatively correlated with trait anxiety levels in adulthood as measured by the STAI (*r*(286) = −.15, *p* = .01; see Fig. 5A). The Caregiver Initiation of Singing (*r*(286) = −.24, *p* < .001; see Fig. 5B) and Attitude Toward Childhood Music Environment (*r*(286) = −.18, *p* = .003; see Fig. 5C) subscales were negatively correlated with the STAI anxiety scores as well. With respect to resilience, the Music@Home–Retrospective general score and all subscales except for the Child Engagement with Music subscale were positively correlated with resilience scores in adulthood as measured by the CD-RISC (General score: *r*(286) = .22, *p* < .001; Caregiver Beliefs: *r*(286) = .14, *p* = .02; Caregiver Initiation of Singing: *r*(286) = .27, *p* < .001; Social Listening Contexts: *r*(286) = .17, *p* = .004; Attitude Toward Childhood Music Environment: *r*(286) = .14, *p* = .02; see Fig. 6).

### Discussion

The goals of Study 2 were threefold. We aimed to confirm the factor solution for the Music@Home–Retrospective scale identified in Study 1, establish test–retest reliability of the scale, and evaluate its associations with measures of adult musicality and mental health. Confirmatory factor analysis indicated a good fit of the model derived from exploratory factor analyses from Study 1 on an independent sample. Further, all five factors and the general factor of the Music@Home–Retrospective scale demonstrated good to excellent test–retest reliability over a period of 4 months. Scores on this scale were also associated with relevant adult (current) behaviors and measures, namely performance on a musical perception task, music usage and enjoyment, and mental health. Together, these results indicate the reliability of the Music@Home–Retrospective Scale and establish preliminary long-term associations between the childhood home musical environment and adult behavior.

## General discussion

While music exposure is a prevalent aspect of individuals’ early home environment, its associations with adult behavior are relatively unknown. Here, we present the Music@Home–Retrospective scale to measure childhood music exposure in adults (18+ years) to better understand these potential long-term associations. Results of both an exploratory and confirmatory factor analysis revealed a bifactor model solution with five constituent factors. Compared to the original Music@Home–Preschool Scale, the Music@Home–Retrospective scale replicates three factors (Caregiver Beliefs, Caregiver Initiation of Singing, and Child Engagement with Music) while also providing two additional factors (Attitude Toward Childhood Home Musical Environment and Social Listening Contexts). This scale has excellent test–retest reliability, and showed associations with adult mental health, music usage, and musicality.

The three factors preserved from the original Music@Home–Preschool Scale in the current scale measure the extent to which (1) participants’ caregivers believed musical training, education, and engagement positively impacted developmental outcomes (Caregiver Beliefs), (2) participants’ caregivers sang to them as children (Caregiver Initiation of Singing), and (3) participants themselves created music (with toys or non-musical items) in middle childhood (Child Engagement with Music). While we included every item from the Music@Home–Preschool Parent Initiation of Musical Behavior factor in our exploratory factor analysis sample, only items indexing the initiation of singing survived our item reduction procedure. This suggests that, although caregivers may report engaging in both singing and music-making with their children (Custodero et al., 2003; Fancourt & Perkins, 2018a; Ilari, 2005; Mendoza & Fausey, 2021; Yan et al., 2021; Young, 2008), the more salient of the two music activities to retrospectively report is caregiver singing. Interestingly, all three of these Music@Home–Retrospective factors are also present on the Music@Home–Infant scale (with Parent Initiation of Singing being its own factor on this scale). This consistency across three versions of this scale suggests that these three dimensions are particularly defining features of the early home musical environment.

Concerning the Caregiver Beliefs and Child Engagement with Music factors, a notable strength of our results was the fact that the scores on these subscales were significantly positively correlated with participants’ MET Melodic scores as adults. This may suggest that the improvement in auditory perception via early musical engagement (Putkinen et al., 2013) might last into adulthood. Alternatively, individuals who report engaging with music in their home might be more likely to receive formal musical training, boosting music perception abilities (Wallentin et al., 2010). In contrast to MET Melodic scores, participants’ MET Rhythmic scores were not related to any subscales of the Music@Home–Retrospective scale. Newborn infants show sensitivity to beats in music (Winkler et al., 2009) and demonstrate adult-like rhythm discrimination abilities as early as 12 months (Hannon & Trehub, 2005a, 2005b). It is thus possible that experiences that fundamentally inform rhythmic (but not necessarily melodic) perception may occur in an earlier developmental time period than the childhood window indexed by the Music@Home–Retrospective scale.

The only factor we did not replicate from the Music@Home–Preschool Scale was the Breadth of Music Exposure factor. This measures the degree to which caregivers exposed their children to an array of musical genres beyond children’s music (e.g., “I was only exposed to "children's music,” which is reverse-scored). “Children’s music” does not include a broad range of musical styles, such as pop, rap, and/or classical music. It is possible that enough participants were not exposed to what would be considered specifically “children’s music” during the 6–12-year window growing up, and thus these questions were unable to capture the breadth of music exposure at these ages. It is also likely easier to self-report exposing your child to various music styles (as in the Preschool version) than retrospectively recalling such exposure. These may account for the fact that this factor and its constituent items did not survive our item reduction procedure.

Exploratory and confirmatory factor analyses also uncovered two additional factors not present in the original Music@Home scales: Attitude Toward Childhood Home Musical Environment, which measures participants’ current attitude and associations with music they were exposed to in childhood (with higher scores representing more positive attitudes), and Social Listening Contexts, which represents the degree to which participants listened to music with others. Interestingly, all items that survived on the Attitude Toward Childhood Home Musical Environment factor were negatively worded (e.g., “I avoid the music my caregivers played me when I was a child”). As we did originally include analogous positively worded questions, it is possible that such items did not capture the same degree of meaningful variability between participants as the negatively worded items. It is interesting to note that this factor showed the lowest ICC value (.64, indicating good reliability). One explanation for this lower value may be that responses to these items are the most variable across time compared to other items. However, we believe that this lower ICC value is attributable to this factor having fewer items (three) than other factors (all consisting of four or five total items). Model results showed that Social Listening Contexts subscale scores were significantly positively associated with adult Social Reward Questionnaire scores over and above the effects of any other Music@Home–Retrospective subscale scores. Our results suggest that the Music@Home–Retrospective scale may enable further investigations into how the early home musical environment relates to adult social behavior.

Although we probed engagement with and attitude toward childhood music during adolescence (i.e., the retrospective attitude items), only items probing adult attitude survived our item reduction procedure. Adolescence is a time in which music plays an important role in identity formation (North & Hargreaves, 1999; North et al., 2000). Therefore, these items were initially included to further investigate the socioemotional effects of the childhood home musical environment through engagement with (or departure from) childhood music during this time. These items ask participants to report how they felt about a past time in their life (childhood) during another past time in their life (adolescence); this nested retrospective-autobiographical reporting may require higher cognitive demands. Thus, one reason they might not have survived item reduction might be difficulty in answering them. Further, as adults have the strongest autobiographical associations with adolescent music (Jakubowski et al., 2020; Rathbone et al., 2017), it is also possible that feelings about one’s childhood home music environment during adolescence are obscured by the memories of adolescent music during that period.

We also identified several associations between the Music@Home–Retrospective scale and adult mental health. Scores on the Caregiver Initiation of Singing subscale scores were significantly negatively correlated with levels of adult trait anxiety. As infant-directed singing is linked to greater child–caregiver connectedness (Fancourt & Perkins, 2018a, 2018b), it is possible that this association represents increased emotional resilience as adults via improved caregiver attachment as children (Gee, 2016). This account is supported by the fact that all Music@Home–Retrospective subscales (with the exception of Child Engagement with Music scores) were related to increased adult resilience as indexed by the CD-RISC-10. We also found that all Music@Home–Retrospective subscales were significantly positively correlated with adult healthy music usage. This set of results suggests that early informal engagement with music scaffolds may improve adult regulatory use of music and so improve resilient behavior. While causal links of these relationships require further testing, these associations motivate future research into the relationship between the childhood home musical environment and adult socioemotional outcomes.

## Limitations

We note several limitations to the current study. First, both studies largely consisted of responding to survey measures, so there is the possibility of participant fatigue. However, we note that each study took, on average, less than one hour to complete. Further, while online studies afford large-scale citizen-science approaches typically not feasible for in-person research, our sample was majority White and based in the US. Future testing may extend to more diverse populations outside of the USA to validate this questionnaire for use in other contexts. It is also important to note that since we drew our samples from participants who had previously completed studies conducted by our lab, our sampling method was nonrandom. The age distribution of our sample was also slightly positively skewed. This is important to note, as age-related differences in question comprehension is a considerable limitation of self-reports (Knäuper et al., 2016). However, Music@Home–Retrospective scores were not correlated with age, suggesting there is no age-related bias present in reporting. Similarly, it is possible that the identified associations between Music@Home–Retrospective scores and adult mental health may represent an inadequacy of retrospective reports for individuals with poorer mental health. Given the retrospective association study design, we cannot currently explicitly isolate or disentangle several factors that may influence observed associations. However, the fact that only two Music@Home–Retrospective subscale scores were significantly associated with STAI scores does not suggest a global relationship between retrospective reports and mental health in the present study. As with any self-report questionnaire, it is also possible that other individual difference measures we did not collect influenced responses on the Music@Home–Retrospective scale or that there are other relevant dimensions of the childhood home musical environment we did not identify. We believe the test–retest reliability of this scale is promising and sufficient for future work investigating the effects of individual differences on this scale, which could also illuminate other relevant features of the childhood home musical environment.

Retrospective reports in general have other additional limitations, such as forgetting or inaccurate reporting (Hardt & Rutter, 2004), highlighted by comparisons between prospective and retrospective reports of childhood experiences (Reuben et al., 2016). We also note that participants’ current attitudes toward their childhood home musical environment may impact their objective reports of this environment. However, Attitude Toward Childhood Home Musical Environment scores are only weakly correlated with scores on other Music@Home–Retrospective dimensions (*r* ranging from .15 to .24) in Study 2 (in contrast to the observed correlations between other subscales). This suggests that current attitudes explain only a small proportion of variance in retrospective reporting on the Music@Home–Retrospective scale. Nonetheless, retrospective reports circumvent the logistical challenges associated with measuring childhood music exposure in longitudinal studies. While these general limitations are likely at play for the Music@Home–Retrospective scale, we believe that it may offer invaluable insight into processes that might otherwise be infeasible to measure prospectively. Finally, in characterizing the childhood home musical environment, it is difficult to disentangle the impacts of informal musical environments from the impacts of formal musical training, such as instrumental instruction. Future studies should pair the Music@Home–Retrospective with a retrospective report of formal musical training during childhood to investigate unique and shared long-term associations these experiences have into adulthood.

## Conclusion

Overall, the Music@Home–Retrospective scale provides a useful validated tool with excellent test–retest reliability to characterize the key features of the early home musical environment in adult samples. As the first measure, to our knowledge, to retrospectively index the childhood home musical environment, we believe that the Music@Home–Retrospective scale is the best assessment currently for measuring the downstream effects of such musical exposure in the absence of lifespan longitudinal studies with that goal. We hope this scale will help improve the understanding of the associations between the childhood home musical environment and adult cognition, behavior, and mental health.

## Data Availability

Music@Home–Retrospective Scale can be found here: https://www.plasticityinneurodevelopmentlab.com/musichome-retrospective and data can be found here: https://osf.io/eq496/

## Appendix 1: Items administered to the Exploratory Factor Analysis Sample

This next survey will ask about musical experiences in your home environment. Please answer each question on a scale from 1 ("Completely Disagree") to 7 ("Completely Agree")

For these first questions, answer in accordance with your experience of music in your household as a child (ages 6-12).

1. My caregivers believed that I should learn to play an instrument.
2. My caregivers believed that music was part of a well-rounded education.
3. My caregivers believed music had an impact on my intelligence.
4. My caregivers thought musical activities were important for learning to communicate.
5. I enjoyed making sounds/interacting with musical instruments (including toy ones).
6. I rarely made music.*
7. I did not use objects to intentionally produce sounds.*
8. I enjoyed toys with musical features.
9. My caregivers sang in playful contexts to/with me often
10. My caregivers sang to/with me in many different situations (e.g. during playtime, with friends and family, in the car).
11. My caregivers were not comfortable singing in public or when others were around.*
12. I made music with my caregivers (including toy instruments) frequently.
13. I did not make music (including toy instruments) more than once or twice per week.*
14. I was exposed to a broad range of musical styles at home (e.g. pop, rap, dance, classical, etc)
15. My caregivers sang all different types of songs to me (e.g. adult songs, traditional folk songs).
16. I was only exposed to "children's music."*
17. My caregivers sang mostly children's songs or lullabies to or with me.*
18. My caregivers played me their favorite music (radio, records, tapes/CDs, etc).
19. My caregivers played me mostly children's music.*
20. My caregivers played music in the house every day.

For these next following questions, please answer in accordance with your experience of music in your household as an adolescent (ages 13-18).

21. I regularly chose to listen to the music my caregivers played me as a child.
22. I avoided the music my caregivers played me when I was a child.*
23. My caregivers played me the same music they played me as a child.

For these next following questions, please answer in accordance with your current attitude towards your experience of music in your childhood household.

24. I associate the music I heard as a child with my caregivers.
25. I have vivid memories involving music my caregivers played me as a child.
26. I have positive memories of the music I heard in the home as a child.
27. I associate music I heard as a child with social contexts (listening with caregivers, siblings, friends).
28. I do not like the music my caregivers played in the home when I was a child.*
29. I have negative memories of the music I heard in the home as a child.*
30. I regularly listen to music my caregivers played me when I was a child.

31. I avoid the music my caregivers played me when I was a child.*. ** = reverse-scored*

## Appendix 2: The Music@Home-Retrospective Scale

This survey will ask about musical experiences in your home environment. Please answer each question on a scale from 1 ("Completely Disagree") to 7 ("Completely Agree"). For these questions, answer in accordance with your experience of music in your household as a child (ages 6-12).

1. My caregivers believed that I should learn to play an instrument.
2. My caregivers believed that music was part of a well-rounded education.
3. My caregivers believed music had an impact on my intelligence.
4. My caregivers thought musical activities were important for learning to communicate.
5. I enjoyed making sounds/interacting with musical instruments (including toy ones).
6. I rarely made music.
7. I did not use objects to intentionally produce sounds.
8. I enjoyed toys with musical features.
9. My caregivers sang in playful contexts to/with me often.
10. My caregivers sang to/with me in many different situations (e.g. during playtime, with friends and family, in the car).
11. My caregivers were not comfortable singing in public or when others were around.
12. I did not make music (including toy instruments) more than once or twice per week.
13. My caregivers sang all different types of songs to me (e.g. adult songs, traditional folk songs).
14. My caregivers played music in the house every day.

## For these next following questions, please answer in accordance with your current attitude towards your experience of music in your childhood household.

15. I associate the music I heard as a child with my caregivers.
16. I have vivid memories involving music my caregivers played me as a child.
17. I associate music I heard as a child with social contexts (listening with caregivers, siblings, friends).
18. I do not like the music my caregivers played in the home when I was a child.
19. I have negative memories of the music I heard in the home as a child.
20. I avoid the music my caregivers played me when I was a child.

### Scoring Instructions and Validated Subscales

* = reverse coded question

**Total Music@Home-Retrospective Score**: Sum of all responses

**Caregiver Beliefs**: 1 + 2 + 3 + 4

**Caregiver Initiation of Singing**: 9 + 10 + 11* + 13

**Attitude towards Childhood Home Musical Environment**: 18* + 19* + 20*

**Social Listening Contexts**: 14 + 15 + 16 + 17

**Child Engagement with Music**: 5 + 6* + 7* + 8 + 12*

## Code availability

Our code for these studies can be found here: https://osf.io/eq496/
